# Use of 3D Printing for the Development of Biodegradable Antiplatelet Materials for Cardiovascular Applications

**DOI:** 10.3390/ph14090921

**Published:** 2021-09-11

**Authors:** Juan Domínguez-Robles, Luis Diaz-Gomez, Emilia Utomo, Tingjun Shen, Camila J. Picco, Carmen Alvarez-Lorenzo, Angel Concheiro, Ryan F. Donnelly, Eneko Larrañeta

**Affiliations:** 1 School of Pharmacy, Queen’s University Belfast, 97 Lisburn Road, Belfast BT9 7BL, UK; j.dominguezrobles@qub.ac.uk (J.D.-R.); eutomo01@qub.ac.uk (E.U.); tshen03@qub.ac.uk (T.S.); c.picco@qub.ac.uk (C.J.P.); r.donnelly@qub.ac.uk (R.F.D.); 2 Departamento de Farmacología, Farmacia y Tecnología Farmacéutica, I+D Farma (GI-1645), Facultad de Farmacia, Health Research Institute of Santiago de Compostela (IDIS), Universidad de Santiago de Compostela, 15782 Santiago de Compostela, Spain; luis.diaz.gomez@usc.es (L.D.-G.); carmen.alvarez.lorenzo@usc.es (C.A.-L.); angel.concheiro@usc.es (A.C.)

**Keywords:** three-dimensional printing, antiplatelet, acetylsalicylic acid, vascular graft

## Abstract

Small-diameter synthetic vascular grafts are required for surgical bypass grafting when there is a lack of suitable autologous vessels due to different reasons, such as previous operations. Thrombosis is the main cause of failure of small-diameter synthetic vascular grafts when used for this revascularization technique. Therefore, the development of biodegradable vascular grafts capable of providing a localized and sustained antithrombotic drug release mark a major step forward in the fight against cardiovascular diseases, which are the leading cause of death globally. The present paper describes the use of an extrusion-based 3D printing technology for the production of biodegradable antiplatelet tubular grafts for cardiovascular applications. For this purpose, acetylsalicylic acid (ASA) was chosen as a model molecule due to its antiplatelet activity. Poly(caprolactone) and ASA were combined for the fabrication and characterization of ASA-loaded tubular grafts. Moreover, rifampicin (RIF) was added to the formulation containing the higher ASA loading, as a model molecule that can be used to prevent vascular prosthesis infections. The produced tubular grafts were fully characterized through multiple techniques and the last step was to evaluate their drug release, antiplatelet and antimicrobial activity and cytocompatibility. The results suggested that these materials were capable of providing a sustained ASA release for periods of up to 2 weeks. Tubular grafts containing 10% (*w*/*w*) of ASA showed lower platelet adhesion onto the surface than the blank and grafts containing 5% (*w*/*w*) of ASA. Moreover, tubular grafts scaffolds containing 1% (*w*/*w*) of RIF were capable of inhibiting the growth of *Staphylococcus aureus*. Finally, the evaluation of the cytocompatibility of the scaffold samples revealed that the incorporation of ASA or RIF into the composition did not compromise cell viability and proliferation at short incubation periods (24 h).

## 1. Introduction

Synthetic polymers were previously used for cardiovascular applications. These applications include the production of vascular grafts, stents or even cardiac valves [1,2,3,4]. Conventional polymers used for this purpose are polytetrafluoroethylene (PTFE) and poly(ethylene terephthalate) (PET) [5,6,7]. These non-biodegradable polymers have shown excellent properties to be used as vascular grafts with diameters larger than 6 mm [5]. However, higher rates of occlusion were reported for small diameter vascular grafts prepared using these types of polymers [5]. In order to solve some of the limitations presented by polymeric materials, drugs can be added to obtain polymeric devices with enhanced properties. Drugs such as antibiotics, anticancer drugs or anticoagulant agents, among many others, were combined with polymers for this purpose [1,5,8,9,10,11,12,13,14,15]. The combination of antibiotics and polymers has been extensively studied for the development of anti-infective materials [16,17,18]. Alternatively, the use of anticoagulant drugs was successfully reported for the development of antithrombotic vascular prosthesis [9,10,19].

Several approaches were described to combine polymeric materials with drugs. These approaches include simple strategies such as surface coating [20] and new strategies such as the use of electrospinning or additive manufacturing for the production of drug loading devices [1,9,19]. Additive manufacturing (also known as 3D printing) has several advantages over other techniques used for the production of medical devices. One of these advantages is the ability to prepare devices adapted to patient’s needs/anatomy [1,21,22].

Additive manufacturing includes a wide range of manufacturing techniques based on the addition of layers of materials to form 3D objects. The mechanism used to add these layers can be based on hot-melt extrusion, photopolymerization or sintering among many others [23,24,25,26,27]. This family of technologies has been widely explored for the production of a wide range of drug delivery systems such as oral tablets, suppositories or implantable devices [16,22,23,24,25,28]. However, the use of additive manufacturing for the development of drug-loaded devices for cardiovascular applications is relatively unexplored.

In the present work, we propose the use of a poly(caprolactone) (PCL) combined with an anticoagulant drug, acetylsalicylic acid (ASA), for the development of 3D printed anticoagulant medical devices. Extrusion-based 3D printing was used to combine PCL and ASA. The drug was incorporated to add anti-platelet properties to the material. The physicochemical properties of the resulting materials were evaluated. Moreover, rifampicin (RIF) was added to the formulation containing the higher ASA loading, as a model molecule that can be used to prevent vascular prosthesis infections. Finally, the antiplatelet activity and cytocompatibility of the 3D printed devices were evaluated. These materials can be used for a wide variety of applications such as the development of vascular grafts or catheters, such as central venous lines.

## 2. Results

### 2.1. Three-Dimensional Printing of PCL-Based Materials Loaded with ASA

**Figure 1:**
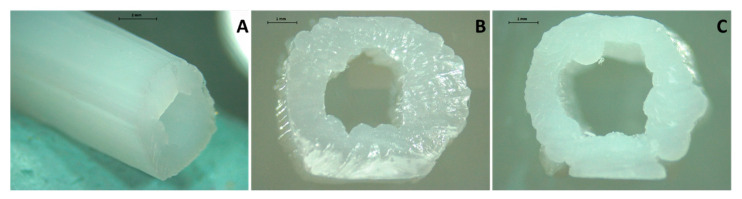
Representative images of a blank tubular graft (**A**) cross-section of a blank tubular graft (**B**) and a tubular graft containing 10% (*w*/*w*) of ASA (**C**) Scale bars: panel **A**: 2 mm; panel **B**,**C**: 1 mm.

Additive manufacturing was used to prepare PCL-based devices loaded with ASA for cardiovascular applications. This drug has antiplatelet properties [29,30,31,32,33] and can be used to prevent blood clot formation on the surface of biomaterials [19,34,35]. Potential applications for the resulting material include the development of vascular grafts or catheters such as arteriovenous fistulas and central venous lines. Accordingly, tubular devices were printed (Figure 1). Three different types of tubular grafts were prepared: a blank (containing no drug) and two different types of grafts containing 5% and 10% (*w*/*w*) of ASA. Figure 1 shows representative pictures of 3D printed tubular devices containing no drug (Figure 1A,B) and 10% (*w*/*w*) of ASA (Figure 1C). Moreover, no drug accumulation or aggregation was observed on the surface of the 3D-printed objects (Figure 1). These results suggest that the drug was well dispersed within the material.

**Figure 2:**
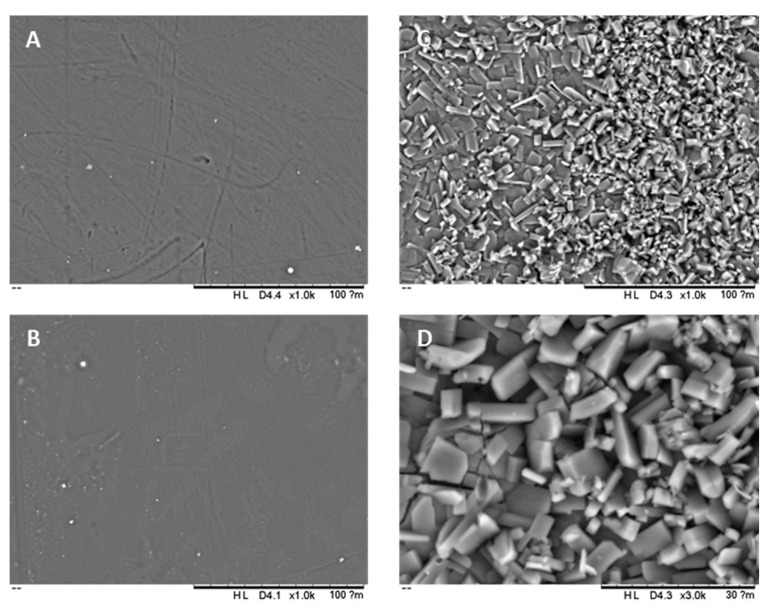
SEM images of the surface of 3D-printed tubular grafts: blank (**A**), 5% (*w*/*w*) ASA (**B**); 10% (*w*/*w*) ASA (**C**); Higher magnification of 10% (*w*/*w*) ASA (**D**).

SEM was used to evaluate the morphology of the surface of the 3D printed tubular grafts (Figure 2). Three-dimensional printed grafts containing no drug and 5% (*w*/*w*) of ASA presented a smooth surface and no drug aggregates were observed (Figure 2A,B). On the other hand, grafts prepared with 10% (*w*/*w*) of ASA had the surface covered by ASA crystals (Figure 2C). Figure 2D shows a higher magnification into the surface of the tubular graft showing aspirin crystals.

### 2.2. Characterisation of Tubular 3D-Printed Tubular Grafts

**Figure 3:**
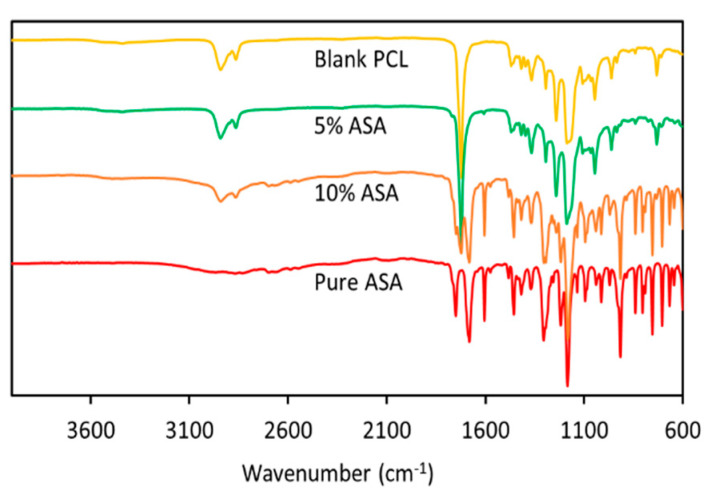
FTIR spectra of ASA and 3D printing tubular grafts containing different ASA content.

FTIR and thermal analysis were used to characterize 3D-printed devices. Figure 3 shows the FTIR analysis of these samples. It can be seen that the blank samples presented the characteristic peaks of PCL: -CH_3_ asymmetric and symmetric stretchings (2943 cm^−1^ and 2869 cm^−1^ respectively) and -C=O stretching (ca. 1720 cm^−1^) [28,36]. On the other hand, the FTIR spectra of ASA showed peaks for the -C=O stretchings (1800–1680 cm^−1^) and the OH stretching (broad peak between 3100 cm^−1^ and 2700 cm^−1^) [37,38,39]. Interestingly, the -C=O stretching peaks of aspirin were evident in the spectra obtained for the samples containing ASA. The intensity of this peak was proportional to ASA concentration in the sample. Finally, there were no new peaks observed when PCL and ASA are combined. This indicates that no chemical reaction took place between these two compounds. The drug is just dispersed within the polymeric matrix.

**Figure 4:**
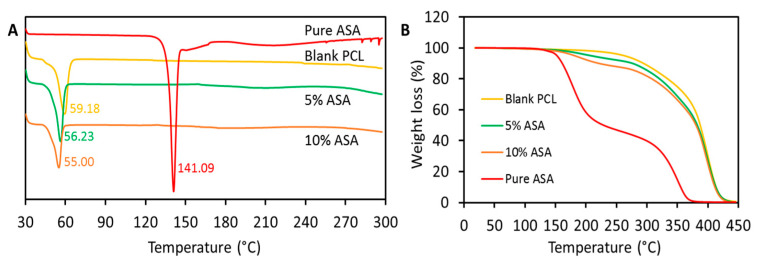
DSC (**A**) and TGA (**B**) analysis for the 3D-printed samples. For DSC curves: exo up.

Figure 4A shows the results obtained for the DSC and the TGA analysis for the 3D-printed devices. The DSC analysis of ASA showed a sharp endothermic peak at 141 °C. This peak can be identified as the melting of ASA [40,41,42]. Interestingly, 3D-printed objects containing ASA did not show the characteristic melting point of the drug. This suggests that the majority of ASA mixed with PCL forming an amorphous dispersion. Interestingly, SEM images showed the presence of crystals on the surface of grafts containing 10% (*w*/*w*) of ASA (Figure 2). It can be concluded that the crystals present on the surface represented a small percentage of ASA as the melting point of the drug cannot be detected when using DSC analysis. On the other hand, blank samples showed the characteristic endothermic peak of PCL melting (ca 60 °C) (Figure 4A) [28]. Samples containing ASA showed the same melting point. However, the melting points decreased with the amount of ASA present in the sample (Figure 4A). This suggests that there is a non-covalent interaction taking place between PCL and ASA. TGA results (Figure 4B) showed that ASA was more thermolabile than PCL as it started degrading at temperatures around 150 °C. This explains that 3D-printed samples containing ASA showed weight losses at lower temperatures than the blank. Moreover, this behavior was more observable for samples containing higher loadings of ASA.

### 2.3. ASA Release Kinetics

**Figure 5:**
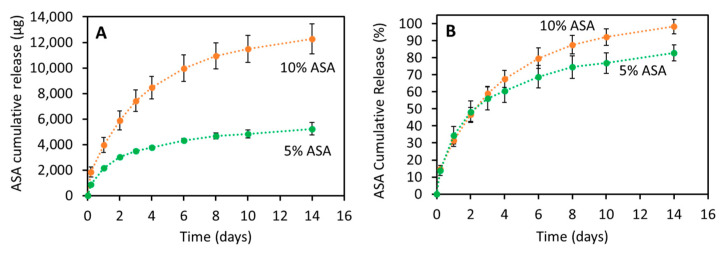
ASA cumulative release from 3D-printed tubular grafts: μg of ASA (**A**) and % of the total ASA cargo (**B**) released as a function of time.

The release of ASA from 3D-printed tubular grafts was evaluated for 2 weeks (Figure 5). Tubular grafts containing 5% and 10% (*w*/*w*) were capable of providing 14 days of ASA release. As expected, grafts containing 10% (*w*/*w*) of ASA released higher amounts of the drug than samples containing lower drug loadings (*p* < 0.05) (Figure 5A). When the results were plotted as the percentage of ASA released as a function of the time (Figure 5B), the release curves obtained for both types of samples were similar. After statistical analysis, it was concluded that there were no significant differences in terms of the percentage of ASA released between both tubular grafts up to day 6 (*p* > 0.05). After day 6 and until the end of the experiments the percentage of ASA released from grafts containing 10% (*w*/*w*) of ASA was significantly greater (*p* < 0.05).

To gain an insight into the release mechanism of ASA from these tubular grafts, the release curves were fitted to different release models (Table 1). The obtained “*n*” values (between 0.45–0.5) for both drug loadings after using the Korsmeyer–Peppas model suggested that Fickian diffusion was responsible for ASA release [43]. This was confirmed after using the Higuchi model and the zero-order model (Table 1). Both curves showed better fitting to the Higuchi model than to the zero-order model, confirming that the diffusion mechanism was governing the drug release process.

### 2.4. Platelet Adhesion Study

**Figure 6:**
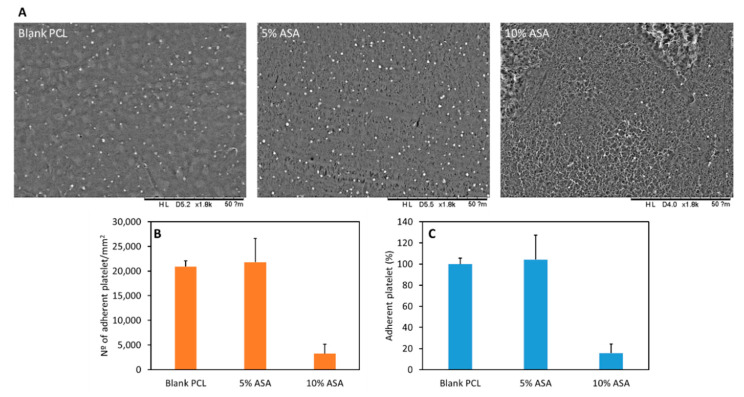
SEM image of the surface of samples containing different amounts of ASA after the platelet adhesion experiment (**A**). Platelet adhesion experiment results for samples containing different ASA loadings. Results are expressed in platelet per mm^2^ (**B**) and percentage of platelets adhered (**C**) using the blank PCL as reference.

Figure 6A shows representative SEM images of platelets adhered to the surface of the 3D-printed materials. Moreover, Figure 6B,C show the results obtained for the platelet adhesion to the surface of 3D-printed samples containing different ASA loadings. Tubular grafts containing 10% (*w*/*w*) of ASA showed lower platelet adhesion onto the surface than the blank and grafts containing 5% (*w*/*w*) of ASA (*p* < 0.05). Samples containing 10% (*w*/*w*) of ASA showed ca. 85% fewer platelets adhered to the surface than other samples (Figure 6B). Alternatively, it can be concluded that there were no significant differences between the blank samples and samples containing 5% (*w*/*w*) of ASA in terms of platelet adhesion (*p* = 0.914).

### 2.5. Three-Dimensional Printing and Characterization of Grafts Containing RIF

**Figure 7:**
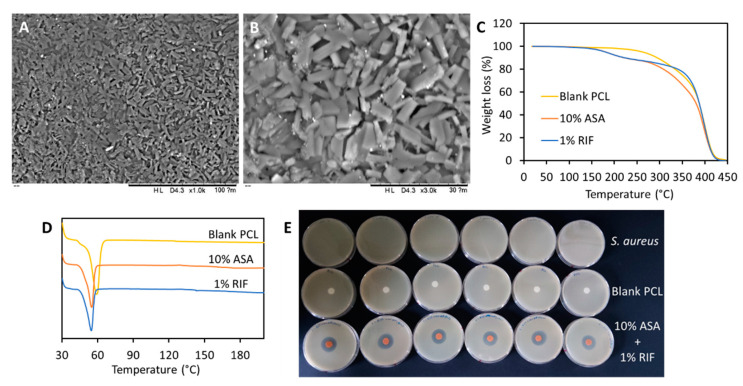
SEM image of 3D-printed samples containing 10% (*w*/*w*) of ASA and 1% (*w*/*w*) RIF (**A**,**B**). TGA (**C**) and DSC (**D**) curves for 3D-printed samples containing different amounts of RIF and ASA. A photograph showing the zones of inhibition obtained for MRSA in MH agar obtained using different 3D-printed samples (**E**).

1% (*w*/*w*) of RIF was added to grafts containing 10% (*w*/*w*) of ASA to evaluate if the resulting samples presented antimicrobial properties. SEM images (Figure 7A,B) of the 3D-printed samples containing 10% (*w*/*w*) of ASA and 1% (*w*/*w*) of RIF showed that the morphology of the surface was similar to that of grafts containing only 10% (*w*/*w*) of ASA. This suggests that RIF was well integrated with the PCL matrix during the printing process. TGA and DSC curves (Figure 7C,D) confirmed this finding. The TGA curve of samples containing 10% (*w*/*w*) of ASA displayed a degradation (weight loss) at lower temperatures than samples containing RIF (Figure 7C). Additionally, DSC (Figure 7D) revealed that the melting point of the samples containing RIF was lower. This suggests that non-covalent interactions between PCL and RIF could be taking place. On the other hand, FTIR of RIF-containing samples was quite similar to the one obtained for samples containing 10% (*w*/*w*) of ASA (data not shown). This could be due to the low RIF loading [1]. Finally, a disk diffusion experiment was performed to evaluate if the 3D-printed samples presented antimicrobial properties. RIF-containing grafts displayed antimicrobial activity against *S. aureus* (Figure 7E). Moreover, a cross-section of a tubular graft containing 10% (*w*/*w*) ASA (10–30 mm in length) was also tested for the inhibitory effect on the same bacterial cultures of *S. aureus* (Figure S1). A small but clear inhibition zone was observed, indicating ASA was also able to promote and therefore enhance the antibacterial activity of the used antibiotic. Similar results were previously reported by using acetylsalicylic acid-loaded electrospun PVP-dextran nanofibers [44].

### 2.6. Cell Proliferation Study

**Figure 8:**
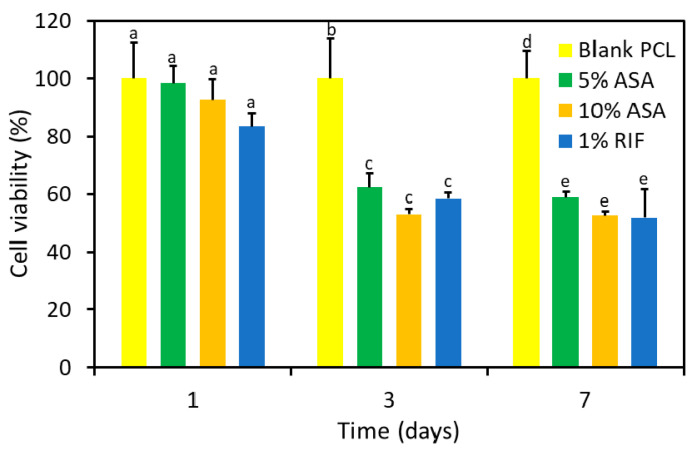
Viability of Balb/3T3 fibroblast after 1, 3 and 7 days in contact with 5% (*w*/*w*) ASA, 10% (*w*/*w*) ASA, 10% (*w*/*w*) ASA and 1% (*w*/*w*) RIF and blank PCL control samples. Different letters denote statistical differences (*p* < 0.05) within the same timepoint.

The cytocompatibility of 5% (*w*/*w*) ASA, 10% (*w*/*w*) ASA, 10% (*w*/*w*) ASA and 1% (*w*/*w*) RIF, and PCL blank grafts was evaluated by direct incubation of the samples with BALB/3T3 fibroblasts (Figure 8). After 24 h in culture, the viability of cells cultured in the presence of disks from all conditions was above 80%, compared to the PCL controls. As the incubation progressed, the accumulation of the released ASA in the culture medium led to a significant decrease in the viability of BALB cells. After 3 days, 5% and 10% (*w*/*w*) ASA samples showed a significant decrease in cell viability (62.3 ± 4.9% and 53.0 ± 1.7%, respectively, compared to the control). Similarly, 10% (*w*/*w*) ASA and 1% (*w*/*w*) RIF samples led to a significant decrease in cell viability to 58.4 ± 4.3%, compared to PCL blank. After 7 days, the viability of cells incubated in the presence of 5% and 10% (*w*/*w*) ASA was similar to the values obtained for each condition after 3 days in culture. A 5% (*w*/*w*) ASA showed a cell viability of 53.1 ± 1.7%, while 10% (*w*/*w*) ASA samples led to a decrease in the cell viability to 52.6 ± 1.5%, compared to the PCL control. The 10% (*w*/*w*) ASA and 1% (*w*/*w*) RIF samples showed viability values (51.8 ± 9.8%) similar to those of 5% and 10% ASA samples, indicating that the incorporation of RIF in the composition had no significant effect on the cytocompatibility of the grafts.

## 3. Discussion

The combination of active molecules, such as drugs or natural compounds, with polymers to obtain materials with advanced properties has been extensively reported in the literature [8,45,46]. Accordingly, the use of drug-loaded polymeric devices for cardiovascular applications was previously reported [1,9,19,47]. One of the key objectives of these materials is to avoid the formation of blood clots within the surface of the biomaterials. For this purpose, several approaches have been described in the literature. These strategies include a surface coating or the use of electrospinning [9,19,48,49,50]. The use of electrospinning was reported extensively for the production of cardiovascular biomaterials, especially for the development of vascular grafts [9,19,48,51].

This work describes the use of an extrusion-based 3D printing technology for the production of biomaterials for cardiovascular applications. This technology was successfully applied to manufacture small-diameter vascular grafts (less than 6 mm diameter) [52]. The use of 3D printing presents some advantages over other technologies such as electrospinning. The main one is its versatility to produce different shapes and objects [1]. Additionally, this type of 3D printing technique does not require the use of solvents like conventional electrospinning [9]. Extrusion-based 3D printing is highly versatile and allows the combination of polymers with drugs in a simple way to obtain materials with advanced properties. In this study, ASA was chosen as a model molecule due to its antiplatelet activity. Accordingly, ASA-loaded materials can be extremely beneficial to prevent the formation of blood clots on the surface of the resulting biomaterial [19]. The use of 3D printing for the development of drug-loaded materials designed for cardiovascular applications is barely explored.

In the present work PCL and ASA were combined using a combination of liquid (low molecular weight, henceforth referred to as L-PCL) and solid (high molecular weight, henceforth referred to as H-PCL) PCL polymers. ASA was first dispersed in the L-PCL and mix it with H-PCL. This mixture was then added to the printer and melted at low temperatures (ca. 60 °C) to print the resulting samples. The use of PCL allows low-temperature 3D printing. Moreover, it is a biodegradable and biocompatible material [53]. The resulting material showed that ASA was well integrated into the material. FTIR results confirmed the presence of ASA within the structure of the 3D-printed samples. Moreover, SEM showed the presence of a certain amount of ASA crystals on the surface of grafts prepared using 10% (*w*/*w*) of ASA. However, DSC analysis did not show any peaks that can be attributed to the presence of significant amounts of crystalline ASA. Moreover, DSC curves suggested that non-covalent interactions between PCL and ASA. Previously published research suggested that the COOH group in ASA forms H-bonds with C=O groups in polymers such as poly(vinyl pyrrolidone) [54]. Considering that PCL has one C=O group per monomer, we can hypothesise that ASA is forming H-bonds with PCL molecules.

The resulting materials were capable of providing a sustained ASA release for periods of up to 2 weeks. Previously reported vascular grafts produced using electrospinning loaded with ASA were capable of providing release of this compound for shorter periods of time (ca. 3 days) [19,55]. The interactions taking place between the polymer and the drug can be responsible for the slower release. The analysis of the release profiles using mathematical models revealed that the release mechanism of ASA from the 3D-printed tubular grafts was Fickian diffusion. Similarly, Del Gaudio et al. analyzed the release profiles of ASA from the electrospun fibers using mathematical models [19]. However, in this study, the results suggested that drug loading had a direct influence on the release mechanism [19]. The release mechanism for vascular grafts prepared using 10% (*w*/*w*) of ASA was a combination of diffusion and matrix degradation (n values obtained for Korsmeyer–Peppas model ranging between 0.5 and 1).

As mentioned before the final objective of these materials is to prevent blood clot formation. Platelet adhesion studies suggested that 3D-printed samples containing 10% (*w*/*w*) of ASA were capable of reducing up to 85% the adhesion of platelets compared to the blank graft (Figure 6). Interestingly, 3D-printed samples containing ASA present higher surface roughness due to the presence of ASA crystals in the surface (Figure 2). Materials with higher surface roughness stimulate platelet adhesion to the surface [56,57]. However, in this case, the effect of the ASA on platelet adhesion overcomes potential platelet activation due to surface roughness. Moreover, these ASA crystals dissolve quickly and the resulting surface presents lower roughness as can be seen in Figure 6A.

There are several examples presented in the literature of ASA-loaded biomaterials. The majority of them are prepared using electrospinning techniques [58,59]. Del Gaudio et al. prepared electrospun grafts loaded with ASA. In this study, grafts containing 10% of ASA showed lower reductions in platelet adherence (ca. 65%) [19]. Moreover, these reductions were obtained after longer periods of time (6 h of incubation with platelet-rich plasma). When samples were incubated for 2 h (such as in the present study) with platelet-rich plasma, electrospun grafts were not capable of providing an anti antiplatelet effect [19]. Interestingly, after shorter periods of time, the presence of aspirin was detrimental as more platelets adhered to the surface of the graft [19]. Accordingly, 3D-printed PCL samples presented superior properties in terms of antiplatelet activity.

Small diameter vascular grafts present several problems including the formation of blood clots [52]. Attempts to overcome this issue include the use of anti-thrombotic drugs into the surface of the material or the development of materials that promote the regeneration of the blood vessel after the degradation of the synthetic graft [9,60]. Accordingly, the addition of ASA and the use of PCL fulfill both objectives. PCL is a biocompatible and biodegradable material [53]. The use of biodegradable polymers loaded with ASA was reported to be highly successful in both: prevent platelet adhesion and promote the endothelialization of biomaterials using an animal model (rabbit) [61].

The use of extrusion-based 3D printing using a heated cartridge allows the simple combination of polymers with several drugs. Accordingly, another drug (RIF) was added to the mixture containing 10% (*w*/*w*) of ASA. RIF is an antibiotic that can be used to prevent bacterial infections of the resulting biomaterial [62]. Interestingly, the addition of RIF affected the thermal properties of the overall material. The melting point of PCL showed a slight reduction (Figure 7D) and the presence of RIF also affected the degradation temperature of the RIF-containing sample (Figure 7C). The effect of RIF on the thermal degradation of polymers has been reported before [1,63]. RIF was reported to form H-bonds with poly(urethane) polymers [1,63]. OH groups of RIF establish H-bonds with the C=O groups of the poly(urethane) chain [63]. Considering the large amount of C=O groups in PCL it can be hypothesized that this phenomenon is taking place in for the combination of PCL and RIF.

The microbiological evaluation suggested that the resulting biomaterials presented antibacterial activity. Infection of prosthetic vascular grafts takes place in up to 6% of patients increasing the morbidity and mortality risk [64]. The incidence of infections is not high, but this complication can put the life of the patient at serious risk. The mortality of prosthetic vascular graft infection can be up to 38% within 2 years after the infection [64]. Accordingly, new anti-infective biomaterials can prevent this issue. Anti-infective 3D-printed materials were previously developed using mainly fused deposition modeling [1,16,17,18]. The main limitation of this particular 3D printing technology is the need to prepare a drug-loaded polymer filament prior to the printing process. The method described here presents some advantages as the drugs and the polymers are directly mixed in a metal cartridge avoiding the extrusion step.

In vitro cytocompatibility testing is critical in the development of biomaterials [65,66,67,68]. Accordingly, this test was carried out for PCL-based vascular grafts described in this study. The evaluation of the cytocompatibility of the grafts revealed that the incorporation of ASA or RIF into the composition did not compromise cell viability and proliferation at short incubation periods (24 h). Moreover, the incorporation of RIF in the composition did not result in a significant decrease in the cytocompatibility, confirming that the grafts prepared with ASA and RIF were endorsed with a remarkable antimicrobial and antiplatelet activity, without negatively affecting the cytocompatibility during short incubation times. The cell viability statistically similar to that of the negative control shown for all conditions after 24 h of incubation confirmed the cytocompatibility of the vascular grafts, in agreement with International Standard ISO 13993-5 [69]. However, the local accumulation of ASA in the incubation media led to a significant decrease in cell viability (ca. 50%, compared to blank PCL controls) after 3 days and 7 days of cell culture. This effect can be attributed to the decrease in the pH of the culture medium due to the released ASA. Several studies have confirmed that a decrease in the extracellular pH led to cell apoptosis [70]. However, previous studies have shown that similar ASA release profiles have no significant effect on the cell cytocompatibility in vivo since the local acidification is mitigated due to the larger blood volume in the implantation site [61,71].

## 4. Materials and Methods

### 4.1. Materials

Poly(caprolactone) (PCL) CAPATM 6506 (Mw = 50,000 Da), henceforth referred to as H-PCL, and PCL CAPATM 2054 (Mw = 550 Da), henceforth referred to as L-PCL were donated by Perstorp (Malmö, Sweden). Acetylsalicylic acid (ASA) was purchased from Sigma-Aldrich (Dorset, UK). Rabbit blood in sodium citrate was provided from Rockland (Reading, UK). Glutaraldehyde 25% EM Grade was obtained from Agar Scientific Ltd. (Essex, UK). Phosphate buffer solution (PBS) was obtained from VWR Chemicals (Solon, OH, USA). Ethanol was provided by Sigma-Aldrich (Dorset, UK). All materials and reagents were used as received. *Staphylococcus aureus* NCTC 10788 was used and incubated overnight at 37 °C in Mueller–Hinton (MH) broth for microbiological studies.

### 4.2. Three-Dimensional Printed Tubular Grafts Design and Manufacture

Tubular grafts were designed using computer-aided design (CAD) software and printed using a 3D bioprinter (Bioscaffolder 3.2, GeSiM) (Radeberg, Germany). The 3D BioScaffolder system was equipped with a 0.5 mm nozzle. The print speed was 10 mm/s, the print temperature used was 60 °C and the layer height and strand distance were 0.25 mm and 0.5 mm, respectively. Moreover, discs (10 mm diameter, 1 mm thickness) were designed and 3D-printed to characterize the 3D-printed materials. For this purpose, the strand distance was set at 0.65 mm, the rest of the parameters remained the same. For the manufacture of these tubular grafts and discs, a mixture of H-PCL (60%) and L-PCL (40%) was selected as the polymer matrix. The PCL mixture was then combined with both 5% and 10% (*w*/*w*) of ASA concentrations or also 10% (*w*/*w*) ASA and 1% (*w*/*w*) RIF by using the SpeedMixer™ DAC 150.1 FVZ-K (Hauschild GmbH & Co. KG, Westfalen, Germany) at 3000 rpm for 5 min and the oven at 80 °C. In brief, ASA was first manually mixed with L-PCL and H-PCL was then added to the mixture, which was placed in the oven for 10 min at 80 °C. Immediately after, the formulation was mixed in the SpeedMixer™ DAC 150.1 FVZ-K at 3000 for 5 min. Both steps were repeated once more and, finally, the formulation was placed into the metal syringe of the 3D bioprinter ready to be printed.

### 4.3. Characterisation of the 3D-Printed Materials

Scanning electronic microscopy (SEM) (Hitachi TM3030; Tokyo, Japan) and a Leica EZ4 D digital microscope (Leica, Wetzlar, Germany) were used to evaluate the morphology of the surface of the 3D-printed materials. The Fourier transform infrared (FTIR) spectra of the 3D-printed materials and the pure ASA were recorded using a Spectrum Two instrument (Perkin Elmer, Waltham, MA, USA) by the attenuated total reflectance (ATR) technique. The spectra were recorded from 4000 cm^−1^ to 600 cm^−1^ with a resolution of 4 cm^−1^, and a total of 32 scans were collected.

Thermal properties of the 3D-printed materials and the pure ASA were evaluated by performing thermogravimetric (TGA) and differential scanning calorimetry (DSC) analysis. TGA was used to establish the degradation temperatures of the 3D-printed materials and the pure ASA, as the formulations were subjected to moderate temperatures (between 60 °C and 80 °C) during the different manufacturing processes. Small fragments of the 3D-printed circles (3 mg and 10 mg) were used. TGA was performed using a Q50 Thermogravimetric analysis (TA instruments, Bellingham, WA, USA). Samples were heated at a rate of 10 °C/min from room temperature to 450 °C under a nitrogen flow rate of 40 mL/min. Moreover, a Q20 differential scanning calorimeter (TA instruments, Bellingham, WA, USA) was used to evaluate if the drug was forming an amorphous dispersion after mixing with the polymer matrix. Thus, once again, small fragments of the 3D-printed circles were used. Scans were run from 30 °C to 300 °C at 10 °C/min under a nitrogen flow rate of 50 mL/min.

### 4.4. ASA Release Kinetics

A release study was performed to calculate the amount of ASA eluting from the resulting 3D-printed tubular grafts. For this purpose, tubular grafts of 5 mm in length containing 5% and 10% (*w*/*w*) ASA were weighed and placed in Falcon tubes containing 5 mL of PBS to maintain sink conditions. Subsequently, these Falcon tubes containing the samples were located in a shaking incubator at 37 °C at 40 rpm. At specific time points, the tubular grafts were removed from the tubes, dried with tissue paper to eliminate excess surface PBS and relocated in new Falcon tubes containing 5 mL of fresh PBS. The release study was conducted for two weeks (*n* = 5). The concentration of ASA was calculated using a UV–visible spectroscopy (FLUOstar Omega Microplate Reader, BMG LABTECH, Ortenberg, Germany). ASA and Salicylic Acid (SA) concentrations were calculated using the absorbance values obtained at two different wavelengths 267 (for SA) and 296 nm (for ASA and SA). Subsequently, the total amount of ASA released was calculated by adding the measured amount of ASA and the equivalent SA quantities as described by Del Gaudio et al. [19]. Korsmeyer–Peppas, Higuchi and Zero-Order models were used to ascertain the release mechanism of ASA as described previously [43].

### 4.5. Platelet Adhesion Study

Rabbit platelet-rich plasma (PRP) was used to measure blood platelet deposition on the surface of the tubular graft. PRP was produced by centrifuging the rabbit blood in sodium citrate (Rockland Immunochemicals, Inc; Pottstown, PA, USA) at 1840 rpm for 15 min [72]. A 200 μL aliquot of PRP was added to the surface of the scaffold samples (discs of 5–6 mm in diameter) in a 96-well plate. Samples were then incubated at 37 °C for 2 h. After this incubation step, samples were rinsed three times with PBS and fixed with a 2.5% glutaraldehyde solution for 2 h. After rinsing three times with PBS, scaffold samples were dehydrated using a series of ethanol solutions (70% and 100%) for 15 min at each step. The samples were then allowed to dry at room temperature for 24 h. Finally, SEM (Hitachi TM3030; Tokyo, Japan) was used to count the adhered blood platelets on the scaffold surface.

### 4.6. Cell Proliferation Study

3D-printed samples (discs of 10 mm diameter and 1 mm thickness) from all conditions (5% (*w*/*w*) ASA, 10% (*w*/*w*) ASA, 10% (*w*/*w*) ASA and 1% (*w*/*w*) RIF and blank PCL control samples) were sterilized in ethanol 70% for 3 h and then incubated in DMEM culture medium (Dulbecco’s modified Eagle´s medium) for 1 h immediately before any in vitro experiment. Scaffolds were placed individually in wells of a 24-well plate and BALB/T3 cells were seeded at a density of 4·10^4^ cells/well and cultured using DMEM complete medium (10% FBS and 1% Pen/Strep). After 1, 3 and 7 days, cell viability was evaluated using a CCK-8 assay (Cell Counting Kit-8; Dojindo Molecular Technologies; Kumamoto, Japan) following the instructions provided by the manufacturer. Briefly, samples were washed with PBS and 500 µL of a CCK-8 working solution (10% *v*/*v* CCK-8 reagent in culture medium) were added to each well and incubated for 2 h at 37 °C. Finally, absorbance was measured at 450 nm (UV Bio-Rad Model 680 microplate reader, Hercules, CA, USA) and cell viability (%) was calculated using non-loaded 3D-printed scaffold samples as negative controls.

### 4.7. Microbiological Assay

Printed discs containing 1% (*w*/*w*) RIF were tested for the inhibitory effect on bacterial cultures of *Staphylococcus aureus* NCTC 10788. A previously described method [16,18] was used for this purpose. In brief, *S. aureus* was incubated overnight at 37 °C in MH broth and 50 µL of this culture was then added to 5 mL of soft MH agar. This mixture was then vortexed and poured on top of an MH agar plate. Printed discs were then placed in the center of the agar plate and incubated for 24 h at 37 °C. The inhibition zone diameters were measured in mm. Further, inoculated plates with *S. aureus* alone and containing PCL discs were also incubated as positive and negative controls, respectively. Additionally, a cross-section of a tubular graft containing 10% (*w*/*w*) of ASA (10–30 mm in length) was also tested for the inhibitory effect on a bacterial culture of *S. aureus*.

### 4.8. Statistical Analysis

All quantitative data were expressed as a mean ± standard deviation. Statistical analysis was performed using a one-way analysis of variance by ANOVA with Tukey’s post-hoc. For the cell proliferation study, one-way ANOVA and Tukey’s multiple comparison post-test were used. Differences were considered significant for *p* < 0.05.

## 5. Conclusions

The development of biodegradable vascular grafts capable of providing a localized and sustained antithrombotic drug release mark a major step forward in the fight against cardiovascular diseases. PCL and ASA were used to prepare biodegradable antithrombotic vascular grafts using an extrusion-based 3D printing technique. The resulting 3D-printed grafts were capable of sustaining ASA release for periods of up to 2 weeks. Vascular grafts with higher ASA concentrations were capable of reducing the adhesion of platelets to the material. Moreover, RIF was combined with ASA and PCL to obtain antimicrobial vascular grafts. These materials were capable of inhibiting the growth of *S. aureus.* Finally, the evaluation of the cytocompatibility of the scaffold samples revealed that the incorporation of ASA or RIF into the composition did not compromise cell viability and proliferation at short incubation periods (24 h).

## Figures and Tables

**Table 1:** Results of fitting the release of ASA from 3D-printed tubular grafts to Korsmeyer–Peppas, Higuchi and Zero-Order models.

ASA (%)	Korsmeyer–Peppas			Higuchi		Zero-Order	
	K_KP_	n	r^2^	K_H_	r^2^	K_ZO_	r^2^
5	0.34 ± 0.01	0.45 ± 0.01	0.9930	0.32 ± 0.01	0.9899	0.14 ± 0.03	0.8820
10	0.32 ± 0.01	0.54 ± 0.02	0.9920	0.33 ± 0.01	0.9974	0.18 ± 0.02	0.9483

## Data Availability

Data is contained within the article and supplementary material.

## Supplementary Materials

The following are available online at https://www.mdpi.com/article/10.3390/ph14090921/s1, Figure S1: A picture showing the zones of inhibition for *S. aureus* in MH agar obtained using different 3D-printed samples.
