# Supplementary material for: Use of 3D Printing for the Development of Biodegradable Antiplatelet Materials for Cardiovascular Applications
Source: Pharmaceuticals (Basel). 2021 Sep 11;14(9):921. doi: 10.3390/ph14090921 (PMC8466262; doi:10.3390/ph14090921)
Supplement: Supplementary file 1 [file pharmaceuticals-14-00921-s001.zip › pharmaceuticals-1348922-supplementary.pdf]

Use of 3D-printing for the development of biodegradable antiplatelet materials for cardiovascular applications.

Juan Domínguez-Robles[1], Luis Diaz-Gomez[2], Emilia Utomo[1], Tingjun Shen[1], Camila J. Picco[1], Carmen Alvarez-Lorenzo[2], Angel Concheiro[2], Ryan F. Donnelly[1], Eneko Larrañeta[1,*]

[1]School of Pharmacy, Queen's University Belfast, 97 Lisburn Road, Belfast, BT9 7BL, UK; j.dominguezrobles@qub.ac.uk (J.D.R.); eutomo01@qub.ac.uk (E.U); tshen03@qub.ac.uk (T.J.S.); c.picco@qub.ac.uk (C.J.P); r.donnelly@qub.ac.uk (R.F.D.); e.larraneta@qub.ac.uk (E.L).
[2]Departamento de Farmacología, Farmacia y Tecnología Farmacéutica, I+D Farma (GI-1645), Facultad de Farmacia, Health Research Institute of Santiago de Compostela (IDIS), Universidad de Santiago de Compostela, 15782 Santiago de Compostela, Spain; luis.diaz.gomez@usc.es (L.D.G.); carmen.alvarez.lorenzo@usc.es (C.A.L.); angel.concheiro@usc.es (A.C.).
*Correspondence: e.larraneta@qub.ac.uk

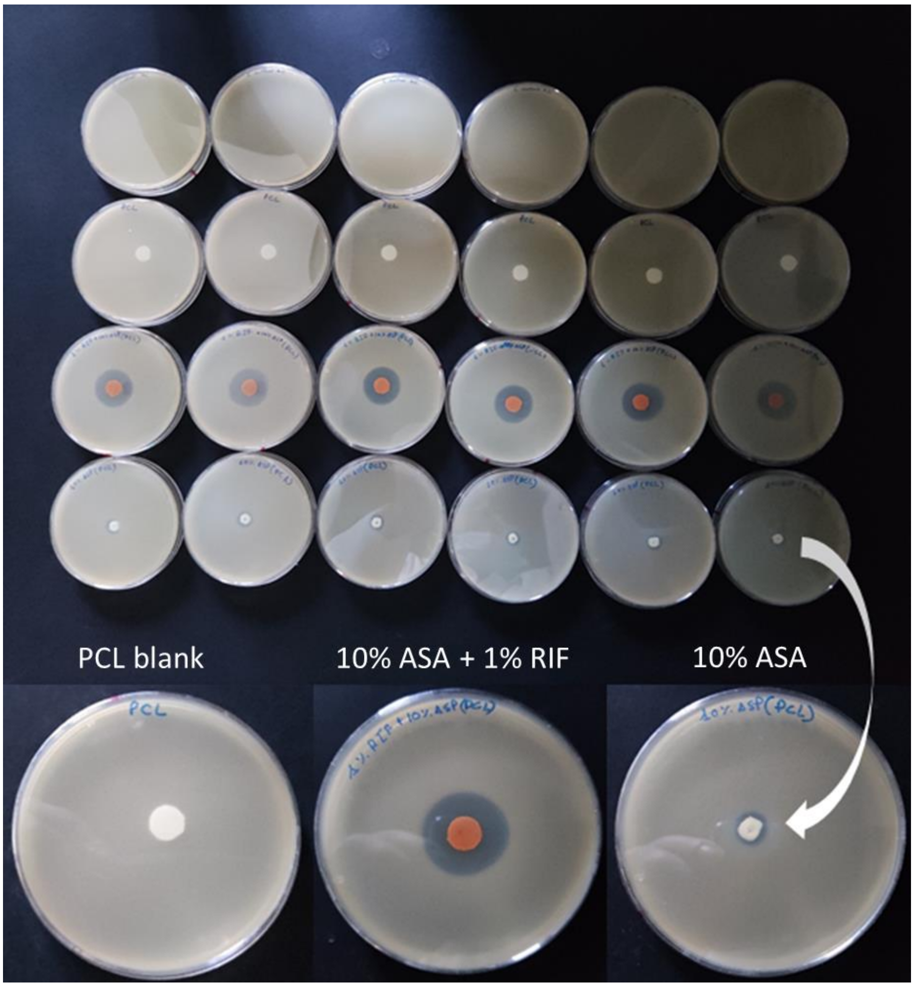

Figure S1. A picture showing the zones of inhibition for *S. aureus* in MH agar obtained using different 3D printed samples.
